# 6-Phenyl­oxane-2,4-dione

**DOI:** 10.1107/S1600536812049781

**Published:** 2012-12-12

**Authors:** Kara A. Slater, Brad Andersh, Edward B. Flint, Gregory M. Ferrence

**Affiliations:** a Mund-Lagowski Department of Chemistry & Biochemistry, Bradley University, Peoria, IL 61625, USA; b Department of Chemistry, University of Iowa, Iowa City, IA 52242, USA; c CB 4160, Department of Chemistry, Illinois State University, Normal, IL 61790, USA

## Abstract

The title compound, C_11_H_10_O_3_, is a phenyl-subsituted dihydro­pyran­dione in which the heterocycle adopts a boat conformation with the phenyl substituent canted 72.14 (5)° relative to the mean plane of the heterocycle.

## Related literature

For the crystal structure of methyl 4-methyl-3,5-dioxo-1-phenyl-2-oxaspiro­[5.5]-4-carboxyl­ate, see: Kirillov *et al.* (2010 ▶) and of *trans*-5,6-diphenyl­perhydro­pyran-2,4-dione, see: de Souza *et al.* (2009 ▶). For the synthesis, see: Andersh *et al.* (2008 ▶). For the biological activity of the title compound and its derivatives, see: Aguiar Amaral *et al.* (2005 ▶); Souza *et al.* (2004 ▶); Tait *et al.* (1997 ▶); Wang *et al.* (1999 ▶). For a description of the Cambridge Structural Database, see: Allen (2002 ▶). A geometry check was performed using *Mogul*, see: Bruno *et al.* (2004 ▶). For puckering parameters, see: Cremer & Pople (1975 ▶).
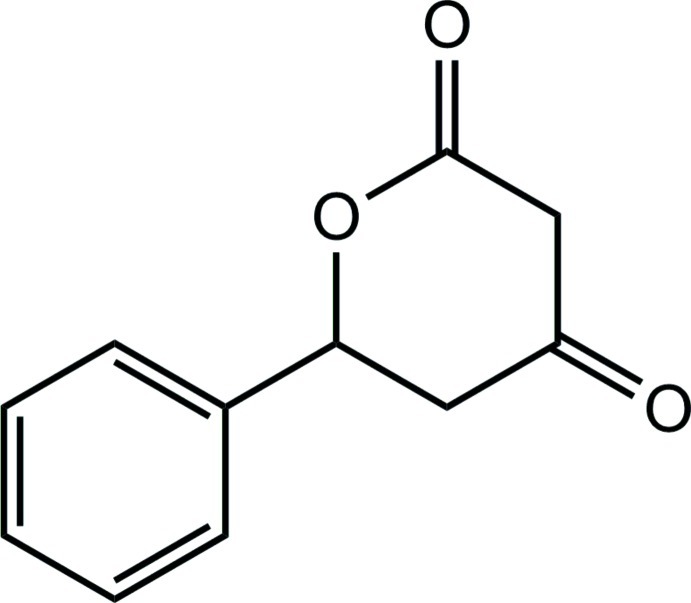


## Experimental

### 

#### Crystal data

C_11_H_10_O_3_
*M* *_r_* = 190.19
Orthorhombic, 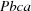

*a* = 16.9888 (6) Å
*b* = 5.4501 (2) Å
*c* = 19.7350 (8) Å
*V* = 1827.28 (12) Å^3^
*Z* = 8
Mo *K*α radiation
μ = 0.10 mm^−1^
*T* = 100 K
0.17 × 0.14 × 0.03 mm

#### Data collection

Bruker APEXII CCD diffractometer
Absorption correction: multi-scan (*SADABS*; Bruker, 2008 ▶) *T*_min_ = 0.662, *T*_max_ = 0.746
17960 measured reflections
1804 independent reflections
1322 reflections with *I* > 2σ(*I*)
*R*_int_ = 0.071

#### Refinement

*R*[*F* ^2^ > 2σ(*F* ^2^)] = 0.047
*wR*(*F* ^2^) = 0.114
*S* = 1.06
1804 reflections
127 parameters
H-atom parameters constrained
Δρ_max_ = 0.29 e Å^−3^
Δρ_min_ = −0.25 e Å^−3^

### 

Data collection: *APEX2* (Bruker, 2008 ▶); cell refinement: *APEX2* and *SAINT* (Bruker, 2008 ▶); data reduction: *SAINT*; program(s) used to solve structure: *SUPERFLIP* (Palatinus & Chapuis, 2007 ▶); program(s) used to refine structure: *SHELXL97* (Sheldrick, 2008 ▶); molecular graphics: *ORTEP-3 for Windows* (Farrugia, 2012 ▶) and *Mercury* (Macrae *et al.*, 2008 ▶); software used to prepare material for publication: *WinGX* (Farrugia, 2012 ▶), *PLATON* (Spek, 2009 ▶) and *publCIF* (Westrip, 2010 ▶).

## Supplementary Material

Crystal structure: contains datablock(s) global, I. DOI: 10.1107/S1600536812049781/bx2433sup1.cif

Structure factors: contains datablock(s) I. DOI: 10.1107/S1600536812049781/bx2433Isup2.hkl

Supplementary material file. DOI: 10.1107/S1600536812049781/bx2433Isup3.cml

Additional supplementary materials:  crystallographic information; 3D view; checkCIF report

## Figures and Tables

**Fig. 1.**
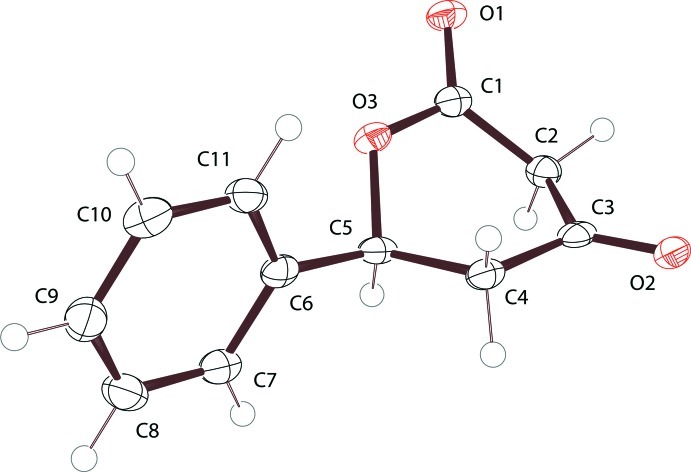
The molecular structure of the compound with the atomic numbering scheme. Displacement ellipsoids are drawn at the 50% probability level.

## supplementary crystallographic information

### Comment

The title compound has a diverse array of biological effects, including reducing sensitivity to pain (Aguiar Amaral *et al.*, 2005) and killing mollusks (Souza *et al.*, 2004). Derivatives of this compound have anti-fungal properties (Wang *et al.*, 1999) and are effective HIV protease inhibitors (Tait *et al.*, 1997).

The molecular structure (Fig. 1.) is the singular moiety in the asymmetric unit. A *Mogul* (Bruno *et al.*, 2004) geometry check showed all non-H bond angles and distances to be normal. Ring puckering analysis of the dihydropyrandione ring using *PLATON* (Spek, 2009; Cremer & Pople, 1975) indicates Φ = 297.5 (2)° and θ = 84.76 (18)° for the O3—C1—C2—C3—C4—C5 ring. These parameters are consistent with a formal conformational assignment close to an idealized B_C2,C5_ boat with C2 at the bow and C5 at the stern. The plane of the phenyl ring attached to C5 may be described as a rudder canted 72.14 (5)° relative to the mean plane of the six core atoms of the heterocycle. The 106.6 (2)° C6—C5—O3 bond angle compared to the 112.8 (2)° C6—C5—C4 bond angle indicates a small steer to said rudder; however, whether it is to port or starboard depends upon which enantiomer is considered.

Based upon a CSD search (Allen, 2002), two structures containing similar lactone ring motifs have been reported in the crystallographic literature. These include the spiro compound methyl 4-methyl-3,5-dioxo-1-phenyl-2-oxaspiro[5.5]-4-carboxylate with CSD refcode IRITIN (Kirillov *et al.*, 2010) and *trans*-5,6-diphenylperhydropyran-2,4-dione with CSD refcode PONVAQ (de Souza *et al.*, 2009). In all three cases the pyran rings adopt the boat conformation.

### Experimental

The title compound 6-(phenyl)-dihydro-2*H*-pyran-2,4-(3*H*)-dione, (also named 5-phenyl-3-oxo-delta-lactone), was prepared by the literature method (Andersh *et al.*, 2008). Benzaldehyde (2 mmol), ethanol (2 ml), ethylacetoacetate (2 mmol), and potassium carbonate (4 mmol) were heated overnight under nitrogen at 318 K. The solution was diluted with ethylacetate, treated with 1 *M* HCl, and the combined organic layer extracts were dried, filtered, concentrated, and purified by flash chromatography.

Crystals suitable for X-Ray analysis were grown by vapor diffusion of pentane into a concentrated solution of the lactone in dichloromethane.

### Refinement

All non-H atoms were refined anisotropically. All H atoms were included in the refinement in the riding-model approximation (C–H = 0.95, 0.99, and 1.00 Å for Ar–H, CH_2_, and CH; *U*_iso_(H) = 1.2Ueq(C).

### Crystal data

**Table:**

C_11_H_10_O_3_	*F*(000) = 800
*M**_r_* = 190.19	*D*_x_ = 1.383 Mg m^−^^3^
Orthorhombic, *P**b**c**a*	Mo *K*α radiation, λ = 0.71073 Å
Hall symbol: -P 2ac 2ab	Cell parameters from 2535 reflections
*a* = 16.9888 (6) Å	θ = 2.4–23.5°
*b* = 5.4501 (2) Å	µ = 0.10 mm^−^^1^
*c* = 19.7350 (8) Å	*T* = 100 K
*V* = 1827.28 (12) Å^3^	Prism, colourless
*Z* = 8	0.17 × 0.14 × 0.03 mm

### Data collection

**Table:**

Bruker APEXII CCD diffractometer	1322 reflections with *I* > 2σ(*I*)
Graphite monochromator	*R*_int_ = 0.071
φ and ω scans	θ_max_ = 26.0°, θ_min_ = 2.1°
Absorption correction: multi-scan (*SADABS*; Bruker, 2008)	*h* = −20→20
*T*_min_ = 0.662, *T*_max_ = 0.746	*k* = −6→6
17960 measured reflections	*l* = −24→24
1804 independent reflections	

### Refinement

**Table:**

Refinement on *F*^2^	Primary atom site location: structure-invariant direct methods
Least-squares matrix: full	Secondary atom site location: difference Fourier map
*R*[*F*^2^ > 2σ(*F*^2^)] = 0.047	Hydrogen site location: inferred from neighbouring sites
*wR*(*F*^2^) = 0.114	H-atom parameters constrained
*S* = 1.06	*w* = 1/[σ^2^(*F*_o_^2^) + (0.039*P*)^2^ + 1.8272*P*] where *P* = (*F*_o_^2^ + 2*F*_c_^2^)/3
1804 reflections	(Δ/σ)_max_ < 0.001
127 parameters	Δρ_max_ = 0.29 e Å^−^^3^
0 restraints	Δρ_min_ = −0.25 e Å^−^^3^

### Special details

**Table:**

Geometry. All s.u.'s (except the s.u. in the dihedral angle between two l.s. planes) are estimated using the full covariance matrix. The cell s.u.'s are taken into account individually in the estimation of s.u.'s in distances, angles and torsion angles; correlations between s.u.'s in cell parameters are only used when they are defined by crystal symmetry. An approximate (isotropic) treatment of cell s.u.'s is used for estimating s.u.'s involving l.s. planes.
Refinement. Refinement of *F*^2^ against ALL reflections. The weighted *R*-factor *wR* and goodness of fit *S* are based on *F*^2^, conventional *R*-factors *R* are based on *F*, with *F* set to zero for negative *F*^2^. The threshold expression of *F*^2^ > 2σ(*F*^2^) is used only for calculating *R*-factors(gt) *etc*. and is not relevant to the choice of reflections for refinement. *R*-factors based on *F*^2^ are statistically about twice as large as those based on *F*, and *R*- factors based on ALL data will be even larger.

### Fractional atomic coordinates and isotropic or equivalent isotropic displacement parameters (Å^2^)

**Table:**

	*x*	*y*	*z*	*U*_iso_*/*U*_eq_	
C1	0.15992 (11)	0.2655 (4)	0.22819 (11)	0.0198 (5)	
C2	0.13487 (12)	0.0646 (4)	0.27556 (11)	0.0208 (5)	
H2A	0.1203	0.1376	0.3198	0.025*	
H2B	0.1799	−0.0474	0.2833	0.025*	
C3	0.06630 (12)	−0.0808 (4)	0.24883 (12)	0.0200 (5)	
C4	0.06699 (12)	−0.1241 (4)	0.17377 (11)	0.0203 (5)	
H4A	0.0222	−0.0357	0.1529	0.024*	
H4B	0.0598	−0.3015	0.1649	0.024*	
C5	0.14301 (12)	−0.0391 (4)	0.14093 (11)	0.0197 (5)	
H5	0.1869	−0.1489	0.1557	0.024*	
C6	0.13875 (12)	−0.0384 (4)	0.06490 (11)	0.0196 (5)	
C7	0.17318 (12)	−0.2284 (4)	0.02863 (12)	0.0240 (5)	
H7	0.2002	−0.3554	0.052	0.029*	
C8	0.16830 (13)	−0.2336 (4)	−0.04141 (12)	0.0289 (6)	
H8	0.1921	−0.364	−0.0659	0.035*	
C9	0.12897 (13)	−0.0501 (4)	−0.07580 (12)	0.0283 (5)	
H9	0.1257	−0.0542	−0.1238	0.034*	
C10	0.09416 (13)	0.1406 (4)	−0.03986 (12)	0.0284 (6)	
H10	0.0672	0.2673	−0.0634	0.034*	
C11	0.09869 (12)	0.1464 (4)	0.03021 (11)	0.0242 (5)	
H11	0.0745	0.2763	0.0546	0.029*	
O1	0.17979 (9)	0.4702 (3)	0.24675 (8)	0.0240 (4)	
O2	0.01417 (8)	−0.1540 (3)	0.28609 (8)	0.0237 (4)	
O3	0.16073 (8)	0.2144 (3)	0.16170 (7)	0.0211 (4)	

### Atomic displacement parameters (Å^2^)

**Table:**

	*U*^11^	*U*^22^	*U*^33^	*U*^12^	*U*^13^	*U*^23^
C1	0.0139 (10)	0.0164 (11)	0.0290 (13)	0.0004 (8)	−0.0010 (9)	−0.0005 (10)
C2	0.0213 (10)	0.0181 (11)	0.0230 (11)	0.0013 (8)	0.0009 (9)	−0.0004 (9)
C3	0.0190 (10)	0.0102 (9)	0.0307 (12)	0.0038 (8)	0.0010 (10)	0.0024 (9)
C4	0.0183 (10)	0.0147 (10)	0.0279 (12)	−0.0013 (9)	0.0001 (9)	−0.0002 (9)
C5	0.0193 (10)	0.0125 (10)	0.0274 (12)	0.0011 (8)	−0.0004 (9)	−0.0019 (9)
C6	0.0151 (10)	0.0168 (10)	0.0269 (12)	−0.0038 (8)	0.0001 (9)	−0.0007 (10)
C7	0.0221 (11)	0.0184 (11)	0.0314 (13)	−0.0003 (9)	0.0002 (9)	−0.0002 (10)
C8	0.0305 (12)	0.0234 (12)	0.0327 (14)	−0.0010 (10)	0.0030 (10)	−0.0079 (11)
C9	0.0332 (13)	0.0285 (13)	0.0232 (12)	−0.0066 (10)	−0.0002 (10)	−0.0006 (11)
C10	0.0315 (13)	0.0210 (12)	0.0326 (14)	−0.0021 (10)	−0.0039 (10)	0.0030 (11)
C11	0.0258 (11)	0.0179 (11)	0.0289 (13)	−0.0008 (9)	0.0017 (10)	−0.0029 (10)
O1	0.0257 (8)	0.0159 (7)	0.0303 (9)	−0.0028 (6)	−0.0007 (7)	−0.0015 (7)
O2	0.0230 (8)	0.0158 (7)	0.0324 (9)	−0.0014 (6)	0.0062 (7)	0.0014 (7)
O3	0.0229 (7)	0.0152 (7)	0.0253 (9)	−0.0048 (6)	−0.0002 (6)	0.0004 (7)

### Geometric parameters (Å, º)

**Table:**

C1—O1	1.222 (3)	C5—H5	1
C1—O3	1.342 (2)	C6—C7	1.388 (3)
C1—C2	1.501 (3)	C6—C11	1.395 (3)
C2—C3	1.505 (3)	C7—C8	1.385 (3)
C2—H2A	0.99	C7—H7	0.95
C2—H2B	0.99	C8—C9	1.381 (3)
C3—O2	1.218 (2)	C8—H8	0.95
C3—C4	1.500 (3)	C9—C10	1.391 (3)
C4—C5	1.517 (3)	C9—H9	0.95
C4—H4A	0.99	C10—C11	1.385 (3)
C4—H4B	0.99	C10—H10	0.95
C5—O3	1.472 (2)	C11—H11	0.95
C5—C6	1.502 (3)		
			
O1—C1—O3	118.69 (19)	C6—C5—H5	109.2
O1—C1—C2	123.9 (2)	C4—C5—H5	109.2
O3—C1—C2	117.43 (18)	C7—C6—C11	119.4 (2)
C1—C2—C3	112.66 (18)	C7—C6—C5	119.53 (19)
C1—C2—H2A	109.1	C11—C6—C5	121.03 (19)
C3—C2—H2A	109.1	C8—C7—C6	120.3 (2)
C1—C2—H2B	109.1	C8—C7—H7	119.8
C3—C2—H2B	109.1	C6—C7—H7	119.8
H2A—C2—H2B	107.8	C9—C8—C7	120.3 (2)
O2—C3—C4	123.38 (19)	C9—C8—H8	119.9
O2—C3—C2	121.6 (2)	C7—C8—H8	119.9
C4—C3—C2	115.03 (18)	C8—C9—C10	119.8 (2)
C3—C4—C5	112.35 (17)	C8—C9—H9	120.1
C3—C4—H4A	109.1	C10—C9—H9	120.1
C5—C4—H4A	109.1	C11—C10—C9	120.2 (2)
C3—C4—H4B	109.1	C11—C10—H10	119.9
C5—C4—H4B	109.1	C9—C10—H10	119.9
H4A—C4—H4B	107.9	C10—C11—C6	120.0 (2)
O3—C5—C6	106.59 (17)	C10—C11—H11	120
O3—C5—C4	109.98 (16)	C6—C11—H11	120
C6—C5—C4	112.73 (17)	C1—O3—C5	117.72 (16)
O3—C5—H5	109.2		
			
O1—C1—C2—C3	139.8 (2)	C11—C6—C7—C8	0.4 (3)
O3—C1—C2—C3	−40.6 (3)	C5—C6—C7—C8	178.5 (2)
C1—C2—C3—O2	−141.94 (19)	C6—C7—C8—C9	−0.1 (3)
C1—C2—C3—C4	37.3 (2)	C7—C8—C9—C10	0.0 (3)
O2—C3—C4—C5	−173.43 (19)	C8—C9—C10—C11	−0.2 (3)
C2—C3—C4—C5	7.4 (2)	C9—C10—C11—C6	0.5 (3)
C3—C4—C5—O3	−50.7 (2)	C7—C6—C11—C10	−0.6 (3)
C3—C4—C5—C6	−169.46 (17)	C5—C6—C11—C10	−178.68 (19)
O3—C5—C6—C7	137.70 (18)	O1—C1—O3—C5	174.97 (17)
C4—C5—C6—C7	−101.5 (2)	C2—C1—O3—C5	−4.6 (3)
O3—C5—C6—C11	−44.2 (2)	C6—C5—O3—C1	173.64 (17)
C4—C5—C6—C11	76.5 (2)	C4—C5—O3—C1	51.1 (2)
